# Intraoperative Fractions of Inspiratory Oxygen Are Associated With Recurrence-Free Survival After Elective Cancer Surgery

**DOI:** 10.3389/fmed.2021.761786

**Published:** 2021-11-26

**Authors:** Sarah Dehne, Verena Spang, Rosa Klotz, Laura Kummer, Samuel Kilian, Katrin Hoffmann, Martin A. Schneider, Thilo Hackert, Markus W. Büchler, Markus A. Weigand, Jan Larmann

**Affiliations:** ^1^Department of Anesthesiology, University Hospital Heidelberg, Heidelberg, Germany; ^2^Department of General, Visceral and Transplantation Surgery, University Hospital Heidelberg, Heidelberg, Germany; ^3^Institute of Medical Biometry, University Heidelberg, Heidelberg, Germany

**Keywords:** supplemental oxygen therapy, postoperative complications, perioperative management, oxygen related effects, recurrence-free survival

## Abstract

**Background:** Choice of the fraction of inspiratory oxygen (FiO_2_) is controversial. The objective of this analysis was to evaluate whether intraoperative FiO_2_ was associated with recurrence-free survival after elective cancer surgery.

**Methods and Analysis:** In this single-center, retrospective study, we analyzed 1,084 patients undergoing elective resection of pancreatic (*n* = 652), colorectal (*n* = 405), or hepatic cancer (*n* = 27) at Heidelberg University Hospital between 2009 and 2016. Intraoperative mean FiO_2_ values were calculated. For unstratified analyses, the study cohort was equally divided into a low- and a high-FiO_2_ group. For cancer-stratified analyses, this division was done within cancer-strata. The primary outcome measure was recurrence-free survival until the last known follow-up. Groups were compared using Kaplan–Meier analysis. A stratified log rank test was used to control for different FiO_2_ levels and survival times between the cancer strata. Cox-regression analyses were used to control for covariates. Sepsis, reoperations, surgical-site infections, and cardiovascular events during hospital stay and overall survival were secondary outcomes.

**Results:** Median FiO_2_ was 40.9% (Q1–Q3, 38.3–42.9) in the low vs. 50.4% (Q1–Q3, 47.4–54.7) in the high-FiO_2_ group. Median follow-up was 3.28 (Q1–Q3, 1.68–4.97) years. Recurrence-free survival was considerable higher in the high-FiO_2_ group (*p* < 0.001). This effect was also confirmed when stratified for the different tumor entities (*p* = 0.007). In colorectal cancer surgery, increased FiO_2_ was independently associated with increased recurrence-free survival. The hazard for the primary outcome decreased by 3.5% with every 1% increase in FiO_2_. The effect was not seen in pancreatic cancer surgery and we did not find differences in any of the secondary endpoints.

**Conclusions:** Until definite evidence from large-scale trials is available and in the absence of relevant clinical conditions warranting specific FiO_2_ values, perioperative care givers should aim for an intraoperative FiO_2_ of 50% in abdominal cancer surgery as this might benefit oncological outcomes.

## Introduction

An increased fraction of inspiratory oxygen (FiO_2_) is frequently used in anesthesia, intensive care, and emergency medicine. A concept of using 80 vs. 30% FiO_2_ has been proposed for the prevention of surgical site infections (SSI) more than 20 years ago (1) and has been evaluated in several subsequent randomized controlled trials (RCTs) (1–3). Based on a systematic review and meta-analysis (4), the WHO and other societies (5–7) issued guidelines advocating the use of 80% FiO_2_ during surgery. These recommendations have caused considerable concern, as the authors of other systematic reviews and meta-analyses arrive at opposing conclusions (8, 9) and because the relevance of potential oxygen-mediated side effects is still under debate (9, 10). In a *post-hoc* analysis of the PROXI trial, administration of 80% oxygen in the perioperative period was associated with increased long-term mortality in patients undergoing cancer surgery (11), and after a median follow-up of 3.9 years, cancer-free survival was significantly reduced in the 80% oxygen group compared with patients randomized to 30% FiO_2_ (12). Outside of clinical trials and before the first WHO recommendation regarding supplemental oxygen (13) was published, intraoperative FiO_2_ was typically used in the range of 30% to more than 90%, and the choice for the actual FiO_2_ did depend on patients' requirements, type of surgical intervention, and anesthesiologist's preferences (14).

Currently, it is unknown whether the choice of higher or lower FiO_2_ in a real-world setting outside of clinical trials is associated with long-term outcomes after cancer surgery. Therefore, we conducted a retrospective and exploratory study to evaluate the association of perioperative FiO_2_ and recurrence-free survival after elective cancer surgery was performed before the WHO recommendation advocating supplemental oxygen for prevention of SSI was published.

## Methods

### Study Design and Population

We performed a retrospective and exploratory study in patients receiving general anesthesia for elective abdominal cancer surgery in the period from January 1, 2009, to December 31, 2016, at the Department of General-, Visceral-, and Transplant Surgery, Heidelberg University Hospital, Heidelberg, Germany. The study protocol conformed to the “Strengthening the Reporting of Observational studies in Epidemiology (STROBE)” guidelines (15), and the principles of the Declaration of Helsinki and was approved by the local Ethics Committee of the Medical Faculty of the Ruprecht-Karls-University, Heidelberg (S-326/2019, August 9, 2019). We assessed whether perioperative FiO_2_ was associated with recurrence-free survival after surgery for three exemplary abdominal cancer entities. Patients with elective surgery of the pancreas, colon or rectum, or liver were included. Subgroup analysis was performed for the three tumor entities. Data were retrieved for all patients ≥ 18 years of age who underwent elective resection of pancreatic (PC), colorectal (CRC,) or hepatic cancer (HC) with R0 (no residual tumor) or R1 (microscopic residual tumor) resection, without distant metastases at the time of surgery, and with follow-up for at least 180 days. Exclusion criteria were intraoperative detection of peritoneal carcinomatosis or if histology analysis could not reveal cancer tissue i.e., after neoadjuvant chemo- or radiotherapy. Patients were also excluded if they suffered familial adenomatous polyposis or did receive home oxygen therapy in the preoperative period.

### Data Collection

Baseline data retrieved from the prospectively maintained electronic pancreas, colorectal, and liver databases of the Department of Surgery at Heidelberg, University Hospital and from the electronic patient file were: demographic data, weight, height, body mass index (BMI), American Society of Anesthesiologists physical status classification (ASA), preexisting diseases including diabetes mellitus and chronic obstructive pulmonary disease (COPD), history of smoking, duration of surgery, need for postoperative ventilation, reintubation, use of epidural anesthesia, intraoperative dose of sufentanil, units of red blood cells (RBC), fresh frozen plasma (FFP), and platelet concentrates (PLT) transfused during surgery and during the entire hospital stay, use of intraoperative radiation therapy (IORT), neoadjuvant and adjuvant radio-, and chemotherapy. For colorectal and pancreatic cancer, tumor site was differentiated as follows: pancreatic head vs. body/tail cancer and rectal vs. colon cancer. Resection margin status, TNM (tumor, node, metastasis) classification, and tumor grading were retrieved from pathology reports. For every patient mean FiO_2_ was calculated on the basis of the FiO_2_ levels documented in the anesthesia protocol at 15-min intervals after induction of anesthesia (first 45 min) until extubation. Furthermore, the measurements of partial pressure of oxygen in arterial blood gases (paO_2_) during surgery were collected. For outcome analyses, intensive care unit and hospital length of stay, date of local recurrence or occurrence of distant metastases, and date and cause of death were collected.

### Outcome Analysis

The primary outcome measure was recurrence-free survival in the period from index surgery until the last known follow-up. Recurrence-free survival was defined as the time from index surgery to the first documented event of local cancer recurrence, newly diagnosed metastases, or death. Computer tomography, abdominal ultrasound, physical examination, and blood sampling were conducted in follow-up examinations at regular intervals or were prompted by new symptoms. If there was no diagnosis of cancer recurrence, new metastases, or death documented, the last date of follow-up or doctor–patient contact with negative findings was recorded. Secondary outcomes were overall survival, sepsis, reoperations due to surgical complications, SSI (superficial incisional, deep incisional, and organ space) and cardiovascular events (myocardial infarction, cerebral infarction, or transitory ischemic attack) during hospital stay were recorded.

### Statistical Analysis

The entire patient cohort was sorted based on ascending mean intraoperative FiO_2_ values and was then divided into two equal sized groups. The low-FiO_2_ group and the high-FiO_2_ group comprised 542 patients each. Descriptive analyses comprised calculation of mean, standard deviation (SD), median, and first and third quartile for continuous variables and absolute and relative proportions for categorical variables. The distribution of categorical variables in the different groups was compared using the chi-square test. Differences in continuous variables were evaluated using the Mann–Whitney *U* test. The primary survival analysis for the prespecified primary endpoint recurrence-free survival was performed using the Kaplan–Meier method (16) and groups were compared by means of the log-rank test (17). As the three tumor entities were not equally distributed over the low- and high-FiO_2_ groups, and because the tumor entities analyzed in our study differed regarding long-term survival rates, we performed an additional analysis to control for tumor entity. First, within each of the three tumor entities, patients were sorted by ascending intraoperative mean FiO_2_ values before patients were equally divided into a low- and high-FiO_2_ group within each entity. Kaplan–Meier curves of these groups were compared within entities. Then patients from the three–tumor entity specific low-FiO_2_ groups were combined into one-entity-controlled low-FiO_2_ group, and patients from the three-tumor entity specific high-FiO_2_ groups were combined in one entity-controlled high-FiO_2_ group. These two groups were compared using a stratified log-rank test with the different tumor entities as strata. Thereafter, the primary outcome was analyzed within the tumor entities PC and CRC by the Cox proportional hazard model (18) in which the effect of mean FiO_2_ on recurrence-free survival adjusted for the following covariates was estimated: gender, age, body mass index, nicotine use, diabetes mellitus, UICC (Tumor classification according to the Union for International Cancer Control) stage, tumor localization, tumor grading, resection margin status, use of epidural anesthesia, intraoperative dose of sufentanil, units of RBC, FFP, and PLT transfused during the entire hospital stay, intraoperative, neoadjuvant and adjuvant radio- and chemotherapy, and laparoscopic surgery in patients with CRC. If fentanyl was used as the opiate instead of sufentanil, the fentanyl dose was multiplied by a factor of 0.1 to calculate the equivalent sufentanil dose (19).

Mean FiO_2_, age, PLT transfusion, intraoperative dose of sufentanil, and BMI are continuous variables, and the other variables are categorial. *P*-values of regression coefficients were obtained by the Wald-Test. Hazard ratios (HRs) estimated from the Cox analysis were reported with corresponding 95% CIs and a two-sided *p* < 0.05 was denoted as considerable. The HC-stratum was too small to fit a covariate-adjusted cox regression. The 1-year and 5-year overall survival was estimated by the Kaplan–Meier method. *P*-value is referred to the stratified log rank test with entities as strata.

Statistical analyses were performed using IBM SPSS Statistics 26.0 (SPSS, Chicago, IL) and Prism 9.0.0 (GraphPad Prism Software, Inc, San Diego, CA).

## Results

**Figure 1:**
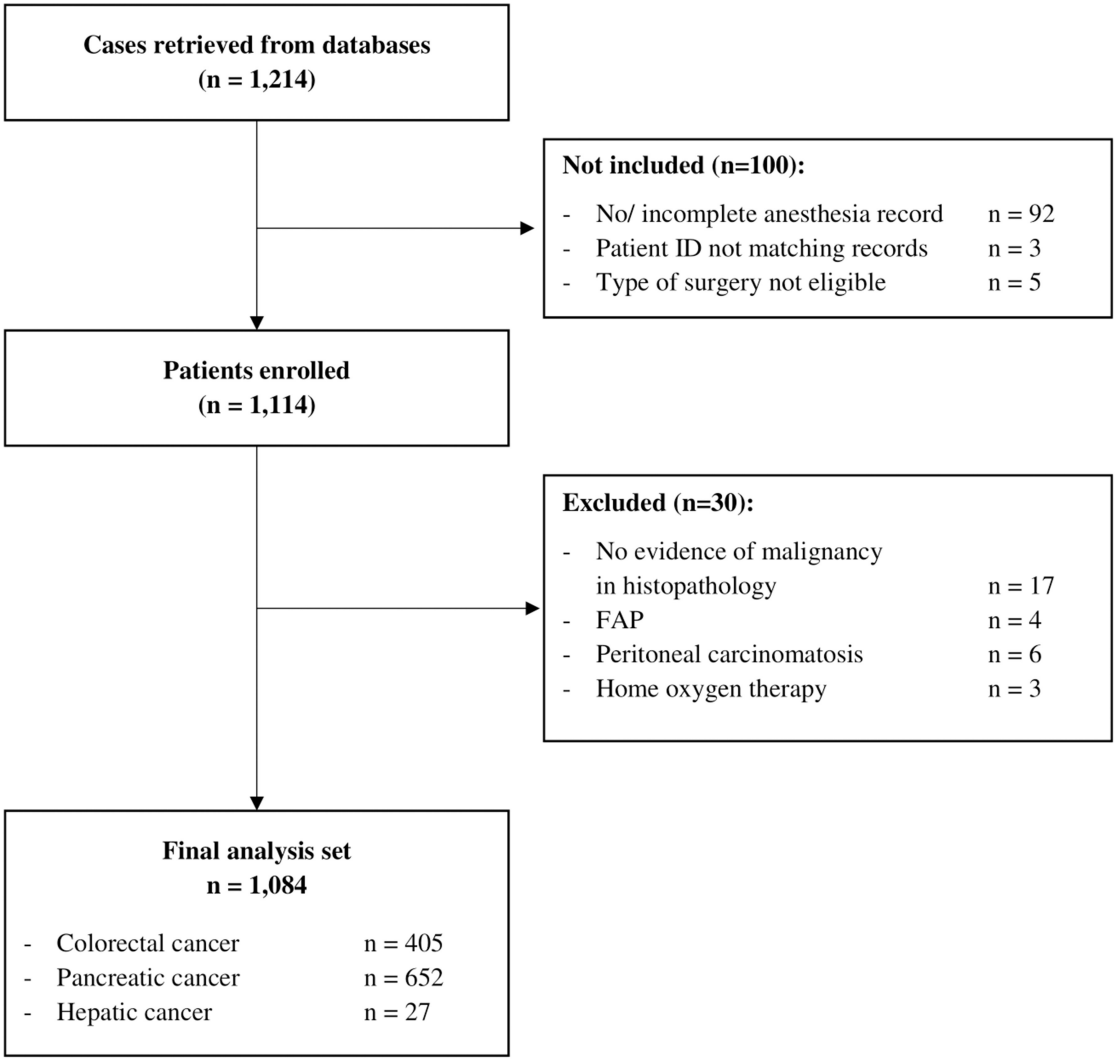
Participant flow chart.

Data from 1,214 patients were retrieved from databases. Datasets from 100 patients could not be assessed for eligibility because of incomplete or missing anesthesia records since the patient ID retrieved from the database did not match with a case in the hospital information system, or because the type of surgery was not eligible, i.e., non-tumor surgery. Considering exclusion criteria, 30 patients had to be excluded. Therefore, 1,084 patients were included in the final analysis set (Figure 1) with a median follow-up of 3.28 (Q1–Q3, 1.68–4.97) years.

### Patient Characteristics

Main clinical and demographical baseline characteristics are presented in Table 1 and the Supplementary Material. A total of 1,084 patients underwent elective PC (*n* = 652), CRC (*n* = 405), or HC surgery (*n* = 27). The mean age was 63 ± 11 years. Male participants were slightly more than female participants. At the time of surgery, the most common cancer stage was UICC 2 and the most common tumor grades were 1 and 2. Around 45% of patients' tumor resection margin status in the pathology report was classified as R1. In the majority of patients with PC, “pancreatic head” was found as the tumor localization; in the majority of patients with CRC “rectum” was found as the tumor localization. Patients received neoadjuvant chemotherapy or neoadjuvant radiotherapy in 15% of cases and 70% received adjuvant chemotherapy. IORT was conducted in 3%. General anesthesia was performed as balanced anesthesia except in four patients. In a small proportion (four patients (0.3%)), fentanyl was used instead of sufentanil and the corresponding equivalent dose was calculated. Intraoperative dose of sufentanil was slightly higher in the low-FiO_2_-groups of patients with CRC and PC (CRC: 88.45 μg ± 45.04 vs. 80.12 μg ± 32.37; low vs. high-FiO_2_, *p* = 0.012; PC: 80.86 μg ± 51.16 vs. 72.22 μg ± 42.85; low vs. high-FiO_2_, *p* = 0.002; HC: 73.21 μg ± 42.23 vs. 66.92 μg ± 27.80; low- vs. high-FiO_2_, *p* = 1.0). Epidural anesthesia was more common in the high-FiO_2_-groups of patients with CRC and PC (CRC: 21(10%) vs. 39 (19%); low- vs. high-FiO_2_*, p* = 0.011; PC: 230 (71%) vs. 258 (79%); low- vs. high-FiO_2_, *p* = 0.011; HC: 10 (71%) vs. 10 (77%), *p* = 0.745). In total, 111 (10.2%) patients were transfused intraoperatively and 284 (26.2%) during their entire hospital stay. There was no difference between the entity-controlled low-FiO_2_ and entity-controlled high-FiO_2_ groups (intraoperative transfusion: 54 (9.9%) vs. 57 (10.5%); entity-controlled low-FiO_2_ vs. entity-controlled high-FiO_2_; *p* = 0.784; transfusions during entire hospital stay: 139 (25.6%) vs. 145 (26.8%); entity-controlled low-FiO_2_ vs. entity-controlled high-FiO_2_; *p* = 0.652).

**Table 1:** Clinical baseline characteristics of the study cohort.

**Variable**	**Analysis set** **(***n*** = 1084)**	**low-FiO_2_** **(***n*** = 542)**	**high-FiO_2_** **(***n*** = 542)**	***p*** **value**
Cancer entity, *n* (%)				
Colorectal cancer (CRC) Pancreatic cancer (PC) Hepatic cancer (HC)	405 (37.4) 652 (60.1) 27 (2.4)	145 (26.8) 391 (72.1) 6 (1.1)	260 (48.0) 261 (48.2) 21 (3.9)	**<0.001**
Age (years), mean ± SD	63.11 ± 10.87	62.17 ± 11.18	64.06 ± 10.47	**0.003**
Male, *n* (%)	614 (57)	305 (50)	309 (50)	0.806
BMI (kg/m^2^), mean ± SD	25.63 ± 4.14, *n* = 1082	25.41 ± 3.72, *n* = 542	25.86 ± 4.52, *n* = 540	0.322
Smokers, *n* (%)				
active previous	217 (20) 109 (10)	99 (18) 61 (11)	118 (22) 48 (9)	0.196
ASA status, *n* (%)				
1–2 3–4	661 (61) 423 (39)	338 (62) 204 (38)	323 (60) 219 (40)	0.350
Diabetes mellitus, *n* (%)	216 (20)	109 (20)	107 (20)	0.879
COPD, *n* (%)	63 (6)	29 (5)	34 (6)	0.516
Duration of surgery (min), mean ± SD	274.81 ± 106.20	298.63 ± 103.75	250.99 ± 103.75	**<0.001**
Intensive care stay, *n* (%) Duration of intensive care stay (d), mean ± SD	522 (48) 16.78 ± 195.63	273 (50) 14.51 ± 155.31	249 (46) 19.27 ± 232.25	0.145 0.296
Postoperative ventilation, *n* (%) Duration of postoperative ventilation (d), mean ± SD	10 (1) 50.60 ± 55.30	4 (1) 69.13 ± 73.26	6 (1) 38.25 ± 42.74	0.525 1.000
Reintubation, *n* (%) Duration of ventilation after reintubation (d), mean ± SD	27 (3) 190.15 ± 131.56	13 (2) 140.92 ± 241.28	14 (3) 239.38 ± 375.98	0.845 0.390
Duration of hospitalization (d), mean ± SD	16.37 ± 13.24	16.33 ± 11.75	16.41 ± 14.58	0.164

*Data are presented as mean ± SD, or as absolute number (percentage). P-values refer to the comparison between low-FiO_2_ vs. high-FiO_2_ patients. Continuous data were compared using the Mann-Whitney U test. Categorical variables were compared using the chi-square test. Boldface indicates p-values < 0.05. FiO_2_, Fraction of inspired oxygen; SD, Standard deviation; BMI, Body mass index; ASA, risk classification according to the American Society of Anesthesiologists; COPD, Chronic obstructive pulmonary disease*.

The average age was higher in the low-FiO_2_ group (64 ± 10.5 vs. 62 ± 11 years, low- vs. high-FiO_2_,*p* = 0.003). For none of the tumor entities, differences with regard to diabetes, history of smoking, or gender were detected. Also, for neither of the tumor entities we observed differences regarding tumor grading status, UICC stage, resection margin status, neoadjuvant, adjuvant therapy, or IORT. In PC surgery, tumor localization in the pancreatic head was more common in the low-FiO_2_ group (81 vs. 72%, *p* = 0.009; low- vs. high-FiO_2_). There was no difference in CRC patients with regard to tumor localization between both groups. In CRC surgery, open surgery was more common in the high-FiO_2_ group (85 vs. 92%, *p* = 0.029; low- vs. high-FiO_2_). PC and HC surgery were always performed as open surgery. Robotic surgery was not performed in our patient cohort. The proportions of the three tumor entities differed between the low and high-FiO_2_ group (PC: 72.1%, CRC: 26.8%, HC: 1.1% vs. PC 48.2%, CRC 48.0%, HC 3.9%, *p* < 0.001; low- vs. high-FiO_2_). The average duration of PC surgery was longer than the duration of CRC and HC surgery (PC:313 min ± 103, CRC 221 min ± 83, HC: 168 min ± 69, *p* < 0.001).

### Observed FiO_2_ and PaO_2_

**Figure 2:**
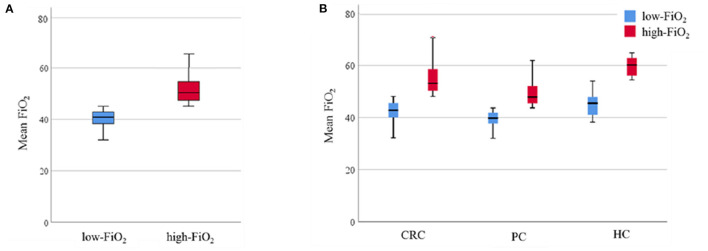
Observed FiO_2_. Data are presented as median (interquartile range). Whiskers indicate the minimum and maximum values. P-values were tested using the non-parametric Mann-Whitney *U* test. **(A)** Intraoperative FiO_2_ levels of the low-FiO_2_ group compared to the high-FiO_2_ group. **(B)** Intraoperative FiO_2_ levels of the low-FiO_2_ groups and the high-FiO_2_ groups of each entity. FiO_2_, Fraction of inspired oxygen; CRC, Colorectal cancer; PC, Pancreatic cancer; HC, Hepatic cancer.

After dividing the cohort into low and high FiO_2_ patients, the cut off value between the two groups was 45.1%, the median of patients' intraoperative FiO_2_ mean [Q1; Q3] in the low-FiO_2_ group was 40.9 [38.3; 42.9] vs. 50.4% [47.4; 54.7] in the high-FiO_2_ group (Figure 2A). In CRC surgery, the calculated cut off value was 48.1%, the median of patients' FiO_2_ means in the low-FiO_2_ group was 42.8 [40.0; 45.6] vs. 53.1% [50.3; 58.6] in the high-FiO_2_ group. In PC surgery, the calculated cut off value was 43.6%, the median of patients' FiO_2_ means in the low-FiO_2_ group was 39.8 [37.7; 41.8] vs. 47.9% [45.5; 52.1] in the high-FiO_2_ group. In HC surgery, the calculated cut off value was 54.0%, the median of patients' FiO_2_ means in the low-FIO_2_ group was 45.5 [41.0; 47.9] vs. 60.2% [56.1; 62.8] in the high-FiO_2_ group (Figure 2B).

**Figure 3:**
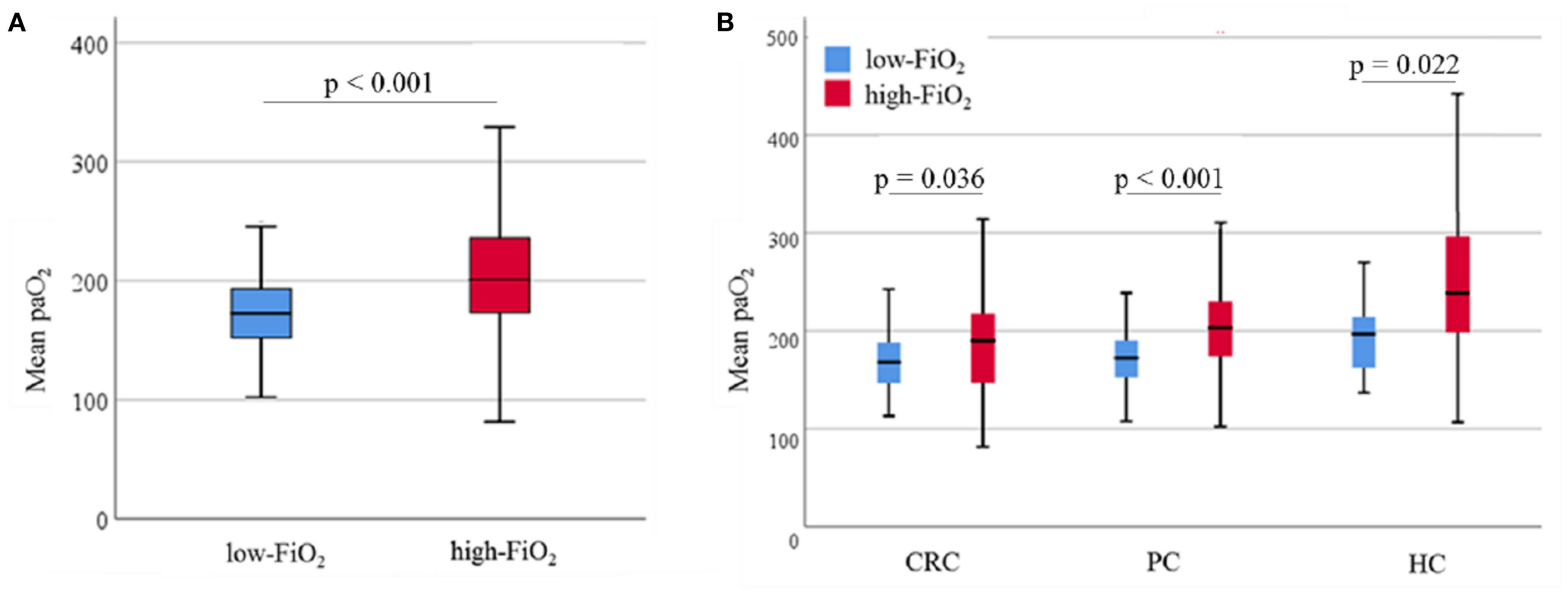
Observed paO_2_. Data are presented as median (interquartile range). Whiskers indicate the minimum and maximum values. *P*-values were tested using the non-parametric Mann-Whitney *U* test. **(A)** Intraoperative mean paO_2_ levels of the low-FiO_2_ group compared to the high-FiO_2_ group. **(B)** Intraoperative mean paO_2_ levels of the low-FiO_2_ groups and the high-FiO_2_ groups of each entity. paO_2_, Arterial oxygen pressure; CRC, Colorectal cancer; PC, Pancreatic cancer; HC, Hepatic cancer.

The mean paO_2_ during surgery was significantly higher in the high-FiO_2_ group (median of patients' intraoperative paO_2_ mean: 172.7 mmHg [152.15; 193.05] vs. 200.7 mmHg [173.2; 235.8], low-vs. high-FiO_2_) (Figure 3A). After stratifying for tumor entities, median paO_2_ values were also significantly lower in the low-FiO_2_ groups compared to the high-FiO_2_ groups (CRC: Median of patients' intraoperative paO_2_ mean: 167.9 mmHg [146.5; 188.0] vs. 190.0 mmHg [147.0; 217.5], low-vs.high-FiO_2_; PC: Median of patients' intraoperative paO_2_ mean: 172.38 mmHg [152.6; 190.3] vs. 203.4 mmHg [174.1; 230.0], low-vs. high-FiO_2_; HC: Median of patients' intraoperative paO_2_ mean: 197.0 mmHg [162.3; 214.3] vs. 238.33 mmHg [198.3; 296.4], low-vs. high-FiO_2_) (Figure 3B).

### Survival Analysis

**Figure 4:**
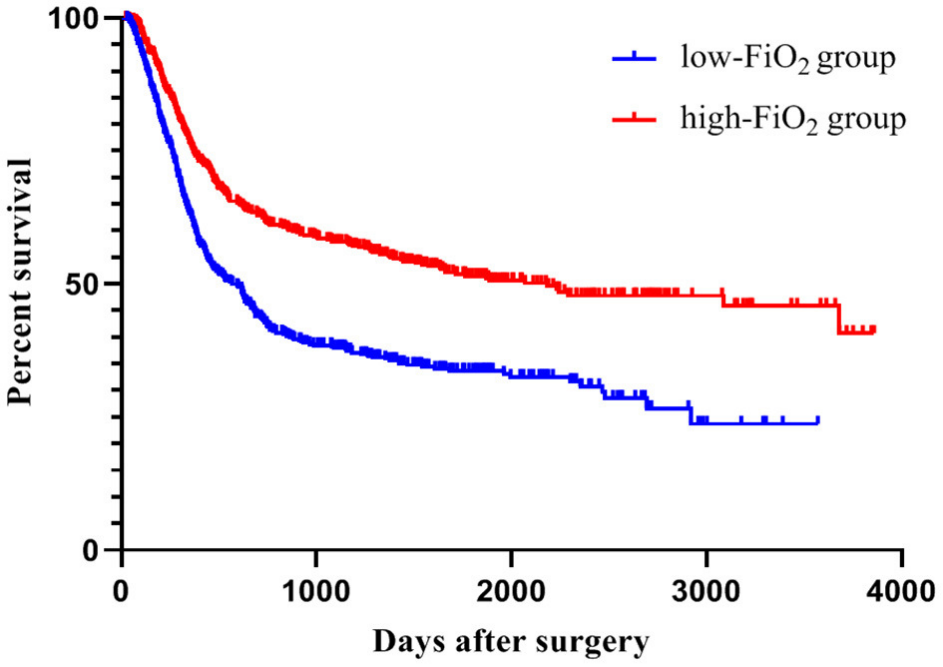
FiO_2_ and recurrence-free survival. Patients were divided into low-FiO_2_ group and high-FiO_2_ group. The p-value was evaluated using the log-rank test (*p* < 0.001). FiO_2_, Fraction of inspired oxygen.

**Figure 5:**
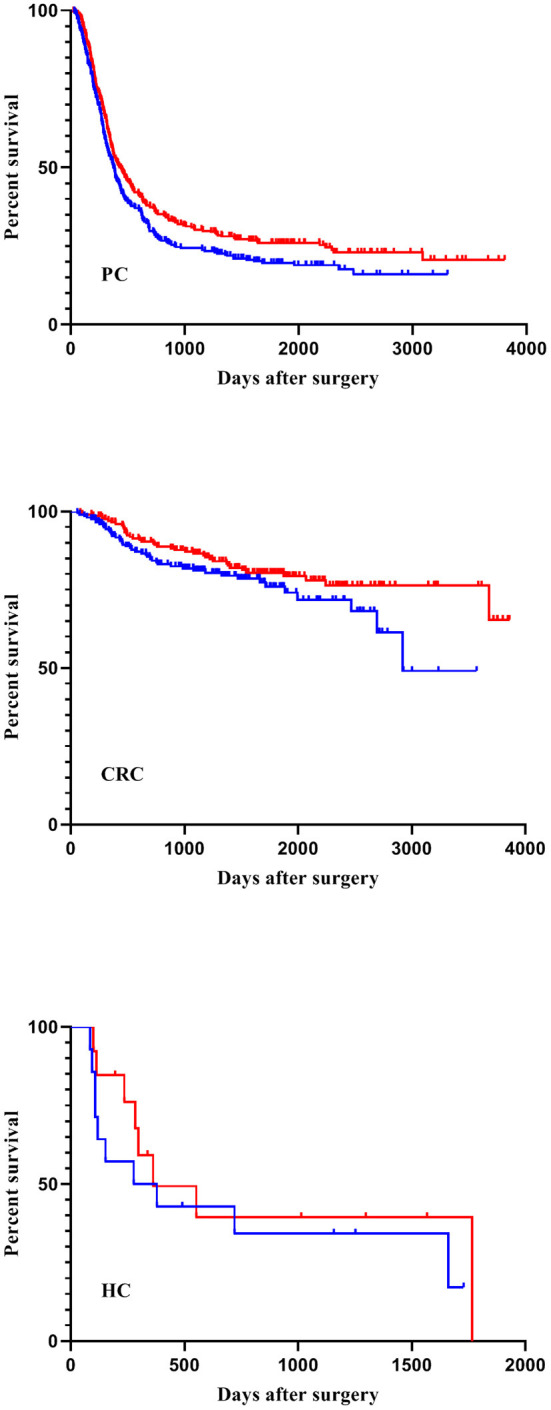
FiO_2_ and recurrence-free survival stratified for tumor entities. Patients were divided into an entity-controlled low-FiO_2_ group and entity-controlled high- FiO_2_ group. A stratified log-rank test with entities as strata was performed (*p* = 0.007). FiO_2_, Fraction of inspired oxygen; CRC, Colorectal cancer; PC, Pancreatic cancer; HC, Hepatic cancer.

In total, cancer recurrence between index surgery and last follow-up was diagnosed in 588 patients (54%). Among those patients, 136 (23.1%) experienced local recurrence and no distant metastases, 309 (52.5%) had distant metastases without local recurrence, and 133 patients (22.6%) suffered from both local relapse and distant metastases. For 10 patients (1.7%), information about the type of recurrence was not available. During the observation period, 402 patients (37.1%) died, mostly (381 cases) due to their malignant disease. Two patients died due to postoperative complications; four patients because of cardiovascular events after hospital discharge. In 15 patients, the cause of death was unknown. Recurrence-free survival was considerably higher in the high-FiO_2_ group (log-rank-test: *p* < 0.001) (Figure 4). This effect was preserved in the entity-controlled groups. Recurrence-free survival was lower in the entity-controlled -low-FiO_2_ group compared to the entity-controlled -high-FiO_2_ group (stratified log rank test: *p* = 0.007) (Figure 5).

The cancer entity itself plays a relevant role in the average survival time of a patient (20–22). Therefore, we performed subgroup analyses for PC and CRC patients respectively using a Cox proportional hazard model, considering factors influencing the observed effect. These analyses revealed an independent association for mean FiO_2_ and recurrence-free survival in patients undergoing CRC surgery (B = −0.035; *p* = 0.025). Recurrence-free survival was longer in patients with higher mean FiO_2_. The hazard ratio (HR) for the variable mean FiO_2_ was 0.965, and thus, HR is 0.965^5^ = 0.837 between two patients whose mean FiO_2_ differ by 5%; per 1% rise of mean FiO_2_ the hazard for the primary outcome decreases by 3.5% (Exp(B): 0.965; 95% CI:0.936–0.996) (Table 2). There was no evidence for an association of FiO_2_ mean with recurrence-free survival in patients undergoing PC surgery (B = -0.01; Exp(B) = 0.990; CI:0.975–1.01; *p* = 0.217) (Table 3). Because the HC group consists of 27 patients only, it was not considered suitable for an individual Cox regression analysis.

**Table 2:** Independent effects of FiO_2_ on recurrence-free survival in patients with CRC.

	**B**	**SE**	**Sig**.	**Exp(B)**	**95% CI for Exp(B)**	
					**Lower**	**Upper**
Female Sex	0.273	0.285	0.339	1.314	0.751	2.299
Age at time of surgery (y)	0.014	0.011	0.217	1.014	0.992	1.037
BMI (kg/m^2^)	0.046	0.031	0.144	1.047	0.984	1.113
No smoking (*reference)*			0.062			
Former smoking	0.254	0.409	0.534	1.290	0.578	2.875
Current smoking	0.684	0.293	**0.019**	1.982	1.117	3.516
Diabetes mellitus	−0.244	0.380	0.521	0.784	0.372	1.650
Dose of sufentanil	0.004	0.003	0.222	1.004	0.998	1.009
Epidural anesthesia	0.300	0.381	0.432	1.350	0.639	2.849
No RBC (*reference)*			0.962			
RBC (1–5 TU)	0.285	0.382	0.456	1.329	0.629	2.809
RBC (6–10 TU)	−13.14	710.33	0.985	0.000	0.000	.
RBC (11–15 TU)	−0.194	1.036	0.852	0.824	0.108	6.276
RBC (>15 TU)	−10.03	882.63	0.991	0.000	0.000	.
PLT (TU)	−10.43	287.61	0.971	0.000	0.000	1.922E + 240
No FFP (*reference)*			0.994			
FFP (1-5 TU)	−0.084	0.778	0.914	0.919	0.200	4.227
FFP (>5 TU)	−12.55	1210.6	0.992	0.000	0.000	.
Laparoscopic surgery	0.009	0.422	0.983	1.009	0.441	2.307
UICC stage 0–1 (*reference)*			**0.000**			
UICC stage 2	1.394	0.522	**0.008**	4.031	1.449	11.213
UICC stage 3	2.440	0.524	**0.000**	11.468	4.104	32.044
Grading G 1–2 (*reference)*			0.062			
No grading due to neoadjuvant therapy	−1.102	0.549	**0.045**	0.332	0.113	0.975
Grading G 3–4	−0.655	0.447	0.143	0.519	0.216	1.247
Resection margin status R1	1.132	0.642	0.078	3.102	0.882	10.907
Rectal cancer	−0.063	0.306	0.837	0.939	0.515	1.711
Neoadjuvant chemotherapy	1.261	0.481	**0.009**	3.529	1.375	9.060
Neoadjuvant radiotherapy	0.279	0.572	0.626	1.321	0.431	4.053
IORT	2.152	1.140	0.059	8.605	0.921	80.377
Adjuvant therapy	0.270	0.299	0.366	1.310	0.729	2.352
Mean FiO_2_	−0.035	0.016	**0.025**	0.965	0.936	0.996

*The p-values of the regression coefficients were calculated using the Wald-Test. Hazard ratios (Exp(B)) estimated from the Cox analysis were reported as relative risks with corresponding 95% CIs. Boldface indicates p-values < 0.05. CRC, Colorectal cancer; B, Coefficient of variable; SE, Standard error; BMI, Body mass index; UICC, Tumor classification according to the Union for International Cancer Control; IORT, intraoperative radiation therapy; FiO_2_, Fraction of inspired oxygen; RBC, Red blood cells; FFP, Fresh frozen plasma; PLT, Platelet concentrates; TU, Transfusion units*.

**Table 3:** Independent effects of FiO_2_ on recurrence-free survival in patients with PC.

	**B**	**SE**	**Sig**.	**Exp(B)**	**95% CI for Exp(B)**	
					**Lower**	**Upper**
Female Sex	−0.014	0.097	0.884	0.986	0.815	1.192
Age at time of surgery (y)	−0.003	0.005	0.491	0.997	0.987	1.006
BMI (kg/m^2^)	−0.018	0.013	0.175	0.983	0.958	1.008
No smoking (*reference)*			0.730			
Former smoking	−0.112	0.171	0.514	0.894	0.639	1.251
Current smoking	−0.069	0.128	0.589	0.933	0.726	1.200
Diabetes mellitus	0.012	0.115	0.915	1.012	0.808	1.268
Dose of sufentanil	0.001	0.001	0.194	1.001	0.999	1.003
Epidural anesthesia	−0.076	0.120	0.527	0.927	0.732	1.173
No RBC (*reference)*			0.722			
RBC (1–5 TU)	0.018	0.122	0.882	1.018	0.802	1.294
RBC (6–10 TU)	0.242	0.278	0.385	1.274	0.738	2.199
RBC (11–15 TU)	−0.526	0.553	0.341	0.591	0.200	1.746
RBC (> 15 TU)	0.217	0.670	0.747	1.242	0.334	4.619
PLT (TU)	0.236	0.225	0.295	1.266	0.814	1.968
No FFP (*reference)*			0.454			
FFP (1–5 TU)	0.218	0.200	0.278	1.243	0.839	1.841
FFP (>5 TU)	0.381	0.425	0.370	1.464	0.637	3.363
UICC stage 2b−3	0.816	0.129	**0.000**	2.260	1.756	2.909
Grading G 1-2 (*reference)*			**0.000**			
No grading due to neoadjuvant therapy	−0.350	0.328	0.286	0.705	0.370	1.340
Grading G 3-4	0.552	0.105	**0.000**	1.737	1.414	2.134
Resection margin status R1	0.264	0.122	**0.030**	1.302	1.026	1.653
Pancreatic head cancer	−0.362	0.119	**0.002**	0.696	0.552	0.878
Neoadjuvant chemotherapy	0.626	0.299	**0.036**	1.871	1.042	3.358
Neoadjuvant radiotherapy	−0.305	0.355	0.391	0.737	0.367	1.479
IORT	0.674	0.349	0.054	1.963	0.990	3.893
Adjuvant therapy	−0.314	0.156	**0.044**	0.731	0.539	0.991
Mean FiO_2_	−0.010	0.008	0.217	0.990	0.975	1.006

*The p-values of the regression coefficients were calculated using the Wald-Test. Hazard ratios (Exp(B)) estimated from the Cox analysis were reported as relative risks with corresponding 95% CIs. Boldface indicates p-values < 0.05. PC, Pancreatic cancer; B, Coefficient of variable; SE, Standard error; BMI, Body mass index; UICC, Tumor classification according to the Union for International Cancer Control; IORT, intraoperative radiation therapy; FiO_2_, Fraction of inspired oxygen; RBC, Red blood cells; FFP, Fresh frozen plasma; PLT, Platelet concentrates; TU, Transfusion units*.

### Secondary Endpoints

We examined 1-year and 5-year survival rates in patients with CRC, PC, and HC separately. In CRC patients, 1-year survival was 100% in both the low- and the high-FiO_2_ group. After 5 years, five out of 203 patients had died (5-year survival 96.2%) in the low-FiO_2_ group, whereas eight out of 202 patients had died in the high-FiO_2_ group (5-year survival 94.6%). In PC patients, 39 out of 326 patients had died after 1 year (1-year survival 87.9%) in the low-FiO_2_ group, whereas in the high-FiO_2_ group 43 out of 326 patients had died (1-year survival 86.7%). After 5 years, 188 patients in the low-FiO_2_ group had died (5-year survival 36.0%) vs. 183 patients in the high-FiO_2_ group (5-year survival 38.5%). In HC patients, 1-year and 5-year survival was 100% in both the low-and the high-FiO_2_ group. Neither 1- nor 5-year survival was different between the entity-controlled low-FiO_2_ and entity-controlled high-FiO_2_ group (*p* = 0.525) (Table 4).

**Table 4:** Secondary outcome analysis.

**Overall survival**	**Analysis set** **(*n =* 1084)**	**Entity-controlled low-FiO_**2**_ (*n =* 543)**	**Entity-controlled high-FiO_**2**_ (*n =* 541)**	***p*** **value**
**(A) Overall Survival entity-controlled low-FiO_2_ group compared to the entity-controlled high-FiO_2_ group**				
**CRC:** Number of patients alive after one year (1-year survival rate in %) Number of patients alive after five years (5-year survival rate in %)	***n =*** **405** 405 (100) 392 (96.8)	***n =*** **203** 203 (100) 198 (97.5)	***n =*** **202** 202 (100) 194 (96.0)	0.525
**PC:** Number of patients alive after one year (1-year survival rate in %) Number of patients alive after five years (5-year survival rate in %)	***n =*** **652** 570 (87.4) 281 (43.1)	***n =*** **326** 287 (88.0) 138 (42.3)	***n =*** **326** 283 (86.8) 143 (43.9)	
**HC:** Number of patients alive after one year (1-year survival rate in %) Number of patients alive after five years (5-year survival rate in %)	***n =*** **27** 27 (100) 27 (100)	***n =*** **14** 14 (100) 14 (100)	***n =*** **13** 13 (100) 13 (100)	
**Secondary outcome**	**Analysis set** **(***n =*** 1084)**	**low-FiO_2_ (*****n =*** **542)**	**high-FiO_2_ (*****n =*** **542)**	***p*** **value**
**(B) Other secondary outcomes low-FiO_2_ group compared to the high-FiO_2_ group**				
Cardiovascular event during hospitalization, *n* (%)	10 (0.9)	5 (0.9)	5 (0.9)	1.000
SSI, *n* (%) Superficial incisional Deep incisional Organ/ space	116 (10.7) 60 (5.5) 4 (0.4) 52 (4.8)	61 (11.2) 31 (5.7) 3 (0.6) 27 (5.0)	55 (10.1) 29 (5.4) 1 (0.2) 25 (5.0)	0.556 0.791 0.316 0.776
Sepsis, *n* (%)	11 (1.0)	5 (0.9)	6 (1.1)	0.762
Reoperation during hospitalization, *n* (%)	76 (7.0)	40 (7.4)	36 (6.6)	0.634

***(A)** 1-year and 5-year overall survival were estimated by the Kaplan Meier method. P-value is referring to the stratified log rank test with entities as strata. **(B)** Categorial variables were compared using the chi-square test. Boldface indicates p-values < 0.05. Occurrence of myocardial infarction, cerebral infarction, or transitory ischemic attack during hospitalization was subsumed as cardiovascular events. FiO_2_, Fraction of inspired oxygen; SSI, surgical site infection; CRC, Colorectal cancer; PC, Pancreatic cancer*.

Cardiovascular events (5 vs. 5, low-vs. high-FiO_2_, *p* = 1) and incidence of sepsis (5 vs. 6, low- vs. high-FiO_2_, *p* = 0.76) did not differ between groups. There was no difference in the rate of SSI (61 vs. 55, low-vs. high-FiO_2_, *p* = 0.556), and the occurrence of reoperations was not different (40 vs. 36, *p* = 0.634, low-vs. high-FiO_2_) (Table 4).

## Discussion

In this retrospective exploratory study, we demonstrated in a real-world setting that higher intraoperative FiO_2_ during abdominal cancer surgery was associated with better recurrence-free survival. In CRC surgery, higher FiO_2_ was independently associated with increased recurrence-free survival. The hazard for the primary outcome decreased by 3.5% with every 1% increase in FiO_2_. In PC surgery, we did not observe a significant effect and we did not find differences in any of the secondary endpoints.

The patient cohort under investigation had undergone surgery before the first WHO recommendation advocating supplemental oxygen for prevention of SSI (13) was published. Therefore, the decision for a specific FiO_2_ was a clinical decision mainly based on the patients' respiratory function and the anesthetists' preference.

Oxygen has known pro- and anticarcinogenic effects. Hyperoxia may not only cause direct cell damage through the generation of reactive oxygen species (ROS) but also mediates cancerous effects (12, 23, 24), such as promotion of proliferation, invasiveness, angiogenesis, and metastasis. Interestingly, ROS generation may be accelerated under hyperoxia and also hypoxia (14). In addition, hypoxia can lead to organ dysfunction, lactic acidosis, cell death (25), and an increased expression of the hypoxia-inducible factor 1 (HIF-1) (26, 27) an intrinsic survival factor of tumor cells. Therefore, hypoxia can induce a vast number of gene products that control neovascularization, cell survival, energy metabolism, intracellular pH, and cell migration and are often associated with increased tumor aggressiveness, therapeutic resistance, and mortality (26, 28–31).

In clinical studies, evidence for FiO_2_-mediated effects associated with long-term outcome after abdominal cancer surgery is sparse and controversial. In a *post-hoc* analysis of the PROXI trial, cancer-free survival was significantly shorter in the high oxygen group (12). In line, after a median follow-up of 2.3 years, a subsequent analysis of the same trial found increased long-term mortality in patients receiving 80% oxygen during cancer surgery (11). Contrarily, authors of other *post-hoc* analysis, combining mortality data from different RCTs, found that there was neither a difference in long-term mortality nor did they find differences in overall survival for patients randomly assigned to 30% vs. 80% FiO_2_ (32, 33). The median follow-up was 12.8 (3.8, 13.6) years in the trial by Greif, 4.3 (3.6, 4.8) years in the trial by Kurz, and 3.2 (0.5, 4.9) years in the study by Jiang (1, 33, 34). This is in line with our finding that overall survival did not differ between high and low FiO_2_ groups with a median follow-up of 3.28 (1.68, 4.97) years. Although FiO_2_ appears to affect cancer recurrence, follow-up in our study might have been too short to observe effects on mortality after recurrence. Also, overall survival did not differ between the two groups investigated here, probably because overall survival depends on a variety of other factors not assessed in this study.

In most prospective studies, comparing different FiO_2_ concentrations, patients were randomized for either 30 or 80% FiO_2_. However, both targets have been accused to cause considerable side effects. Outside of clinical trials and before guidelines advocating high levels of supplemental oxygen were published, most anesthetists chose moderate FiO_2_ levels between the high and low extremes. As a result, in our retrospective study, mean FiO_2_ levels in the low- and high-FiO_2_ groups were closer together than in other studies. Importantly, our institutional standards suggest avoiding not only hypoxia but also hyperoxia as both conditions might cause adverse events.

Pro and anticarcinogenic effects of oxygen might explain why, on one hand, 80% FiO_2_ has adverse effects over 30% FiO_2_ in some studies, but on the other hand, in our study, FiO_2_ in the range of 50% compared with lower FiO_2_ concentrations was beneficial with regard to recurrence-free survival. It is conceivable that the optimal FiO_2_ for tumor surgery patients is neither met with an FiO_2_ of 30% nor 80%. In fact, the association of FiO_2_ or paO_2_ respectively and recurrence-free survival may follow a V-shaped curve.

Oxygen related effects in tumor biology may also differ for distinct types of tumors. The association of tumor-associated transcription factors and tumor progress varies among different tumor entities (26). We included patients with CRC, PC, and HC. We demonstrated an independent association of FiO_2_ with recurrence-free survival for CRC surgery patients but not for patients undergoing PC surgery. Interestingly, Podolyak et al. and Jiang et al. who did not find differences regarding cancer-free survival investigated only patients with elective colectomy in their *post-hoc* analysis, including RCTs (32, 33). In the *post-hoc* analysis of the PROXI trial, about half the patients underwent colorectal surgery (12). A subgroup analysis for non-colorectal procedures was not conducted, although cancer histology was considered (12).

Additional adverse effects of high-FiO_2_ affecting the cardiovascular and respiratory systems have been reported in the literature. Hyperoxia induces increased peripheral vascular resistance, promotes reduced cardiac output, and mediates coronary vasoconstriction (14, 35). A *post-hoc* analysis of the PROXI-trial showed that patients with a FiO_2_ of 80% had a significantly increased risk of myocardial infarction, acute coronary syndrome, or death (36). Contrary, the authors of a systematic review and meta-analysis concluded, based on 17 RCTs and two non-randomized studies, that perioperative supplemental oxygen was not associated with relevant complications (10). Consistent with this report, our analysis did not reveal differences for secondary cardiovascular endpoints or redo surgery.

WHO, ACS (American College of Surgeons), and CDC (Centers for Disease Control and Prevention) recommends supplemental oxygen with the aim to prevent SSI. In our study, SSI did not differ between the high- and low-FiO_2_ groups. One could argue that the FiO_2_ in the high-FiO_2_ group was not sufficiently high to prevent SSI. However, the incidence of SSI reported in this study is lower than reported in most of the RCTs testing different oxygen levels in abdominal cancer surgery patients.

Our study has some limitations that need to be addressed. We performed a retrospective, single-center study with a limited set of exemplary tumor entities. The number of individuals included in the analysis was limited by the availability of digitalized anesthesia records, and only patients recruited in the surgical databases could be analyzed. The choice of FiO_2_ values was not standardized and the patients were equally split into the low- and high-FiO_2_ groups. The patients' intraoperative FiO_2_ levels were compared by the calculation of the individual mean FiO_2_, but the duration of exposure and individual dose during surgery were not included in this calculation. A consequence of the retrospective nature of our study is that we do not have full control over confounders. As tumor entity itself affects outcome, and we conducted an analysis stratified for entities and performed a cox regression analysis to control for confounders. However, we cannot fully exclude, that additional confounders affected findings reported in this study.

In conclusion, we demonstrate that, within a moderate range of FiO_2_, higher FiO_2_ during abdominal cancer surgery was associated with longer recurrence-free survival. In colorectal cancer surgery, increased FiO_2_ was independently associated with increased recurrence-free survival. The findings will be instrumental for designing prospective studies delineating effects of certain FiO_2_ on cancer development and progression for specific patient populations. Until definite evidence from large-scale randomized controlled trials is available, anesthesiologist, in the absence of relevant clinical conditions warranting specific FiO_2_ values, should aim for an intraoperative FiO_2_ of 50% in abdominal cancer surgery as this might benefit oncological outcome.

## Data Availability Statement

The data analyzed in this study is subject to the following licenses/restrictions: Datenschutzgrundverordnung. Requests to access these datasets should be directed to jan.larmann@med.uni-heidelberg.de.

## Ethics Statement

The studies involving human participants were reviewed and approved by Ethics Committee of the Medical Faculty of the Ruprecht-Karls-University, Heidelberg. Written informed consent for participation was not required for this study in accordance with the national legislation and the institutional requirements.

## Author Contributions

SD, VS, RK, and JL designed research and study protocol. SD, VS, LK, SK, and JL formal analysis. JL, SD, and VS statistical analysis. SK methodology and statistical consultation. SD, VS, RK, LK, KH, MS, TH, MB, and MW contributed to the data curation. SD, VS, and JL wrote the first draft of the manuscript. All authors critically reviewed and revised the manuscript and approved the final work.

## Funding

This study was funded by institutional resources from the Departments of Anesthesiology and General, Visceral and Transplant Surgery. The position of LK was funded by the German Research Foundation (LA 2343/7-1) to JL. This research did not receive any other specific grants from funding agencies in the public, commercial, or not-for-profit sectors.

## Conflict of Interest

The authors declare that the research was conducted in the absence of any commercial or financial relationships that could be construed as a potential conflict of interest.

## Publisher's Note

All claims expressed in this article are solely those of the authors and do not necessarily represent those of their affiliated organizations, or those of the publisher, the editors and the reviewers. Any product that may be evaluated in this article, or claim that may be made by its manufacturer, is not guaranteed or endorsed by the publisher.

## Abbreviations

ASA: American Society of Anesthesiologists physical status classification
B: Coefficient of variable
BMI: Body Mass Index
COPD: Chronic Obstructive Pulmonary Disease
CI: Confidence Intervals
CRC: Colorectal cancer
FAP: Familial Adenomatous Polyposis
FiO_2_: Fraction of inspiratory oxygen
FFP: Fresh frozen plasma
HC: Hepatic cancer
HIF-1: Hypoxia-inducible factor 1
HR: Hazard Ratio
IORT: IntraOperative Radiation Therapy
paO_2_: Arterial oxygen pressure
PC: Pancreatic cancer
PLT: Platelet concentrates
Q: Quartile
R 0: No residual tumor
R 1: Microscopic residual tumor
RBC: Red blood cells
RCT: Randomized controlled trial
ROS: Reactive Oxygen Species
SD: Standard Deviation
SE: Standard Error
SSI: Surgical Site Infetions
TNM, Tumor, Node: Metastasis
TU: Transfusion units
UICC: Tumor classification according to the Union for International Cancer Control
WHO: World-Health-Organization.

## References

[1] Greif R Akça O Horn EP Kurz A Sessler DI. Supplemental perioperative oxygen to reduce the incidence of surgical-wound infection. N Engl J Med. (2000) 342:161–7. 10.1056/NEJM200001203420303 10639541

[2] Qadan M Akça O Mahid SS Hornung CA Polk HC. Perioperative supplemental oxygen therapy and surgical site infection: a meta-analysis of randomized controlled trials. Arch Surg. (2009) 144:359–66. 10.1001/archsurg.2009.1 19380650

[3] Pryor KO Fahey TJ Lien CA Goldstein PA. Surgical site infection and the routine use of perioperative hyperoxia in a general surgical population: a randomized controlled trial. Jama. (2004) 291:79–87. 10.1001/jama.291.1.79 14709579

[4] de Jonge S Egger M Latif A Loke YK Berenholtz S Boermeester M. Effectiveness of 80% vs 30-35% fraction of inspired oxygen in patients undergoing surgery: an updated systematic review and meta-analysis. Br J Anaesth. (2019) 122:325–34. 10.1016/j.bja.2018.11.024 30770050

[5] Ban KA Minei JP Laronga C Harbrecht BG Jensen EH Fry DE. American college of surgeons and surgical infection society: surgical site infection guidelines, 2016 update. J Am Coll Surg. (2017) 224:59–74. 10.1016/j.jamcollsurg.2016.10.029 27915053

[6] Berríos-Torres SI Umscheid CA Bratzler DW Leas B Stone EC Kelz RR. Centers for disease control and prevention guideline for the prevention of surgical site infection, 2017. JAMA Surg. (2017) 152:784–91. 10.1001/jamasurg.2017.0904 28467526

[7] WorldHealthOrganization. Global Guidelines for the Prevention of Surgical Site Infection. © World Health Organization (2018). 30689333

[8] Cohen B Schacham YN Ruetzler K Ahuja S Yang D Mascha EJ. Effect of intraoperative hyperoxia on the incidence of surgical site infections: a meta-analysis. Br J Anaesth. (2018) 120:1176–86. 10.1016/j.bja.2018.02.027 29793584

[9] Smith BK Roberts RH Frizelle FA. O(2) No Longer the Go(2): a systematic review and meta-analysis comparing the effects of giving perioperative oxygen therapy of 30% FiO(2) to 80% FiO(2) on surgical site infection and mortality. World J Surg. (2020) 44:69–77. 10.1007/s00268-019-05224-3 31605182

[10] Mattishent K Thavarajah M Sinha A Peel A Egger M Solomkin J. Safety of 80% vs 30-35% fraction of inspired oxygen in patients undergoing surgery: a systematic review and meta-analysis. Br J Anaesth. (2019) 122:311–24. 10.1016/j.bja.2018.11.026 30770049

[11] Meyhoff CS Jorgensen LN Wetterslev J Christensen KB Rasmussen LS. Increased long-term mortality after a high perioperative inspiratory oxygen fraction during abdominal surgery: follow-up of a randomized clinical trial. Anesth Analg. (2012) 115:849–54. 10.1213/ANE.0b013e3182652a51 22798533

[12] Meyhoff CS Jorgensen LN Wetterslev J Siersma VD Rasmussen LS. Risk of new or recurrent cancer after a high perioperative inspiratory oxygen fraction during abdominal surgery. Br J Anaesth. (2014) 113:i74–81. 10.1093/bja/aeu110 24860156

[13] WorldHealthOrganization. Global Guidelines for the Prevention of Surgical Site Infection. Geneva, (2016).

[14] Martin DS Grocott MPIII. Oxygen therapy in anaesthesia: the yin and yang of O2. Br J Anaesth. (2013) 111:867–71. 10.1093/bja/aet291 24233308

[15] Cuschieri S. The STROBE guidelines. Saudi J Anaesth. (2019) 13:S31–s4. 10.4103/sja.SJA_543_18 30930717 PMC6398292

[16] Kaplan EL Meier P. Nonparametric estimation from incomplete observations. J Am Stat Assoc. (1958) 53:457–81. 10.1080/01621459.1958.10501452

[17] Mantel N. Evaluation of survival data and two new rank order statistics arising in its consideration. Cancer Chemother Rep. (1966) 50:163–70. 5910392

[18] Cox DR. Regression Models and Life-Tables. J R Stat Soc B. (1972) 34:187–220. 10.1111/j.2517-6161.1972.tb00899.x

[19] Nielsen S Degenhardt L Hoban B Gisev N. A synthesis of oral morphine equivalents (OME) for opioid utilisation studies. Pharmacoepidemiol Drug Saf. (2016) 25:733–7. 10.1002/pds.3945 26693665

[20] Klaiber U Schnaidt ES Hinz U Gaida MM Heger U Hank T. Prognostic factors of survival after neoadjuvant treatment and resection for initially unresectable pancreatic cancer. Ann Surg. (2021) 273:154–62. 10.1097/SLA.0000000000003270 30921051

[21] Strobel O Hank T Hinz U Bergmann F Schneider L Springfeld C. Pancreatic cancer surgery: the new r-status counts. Ann Surg. (2017) 265:565–73. 10.1097/SLA.0000000000001731 27918310

[22] Ulrich CM Gigic B Böhm J Ose J Viskochil R Schneider M. The colocare study: a paradigm of transdisciplinary science in colorectal cancer outcomes. Cancer Epidemiol Biomarkers Prev. (2019) 28:591–601. 10.1158/1055-9965.EPI-18-0773 30523039 PMC6420345

[23] Selvendiran K Kuppusamy ML Ahmed S Bratasz A Meenakshisundaram G Rivera BK. Oxygenation inhibits ovarian tumor growth by downregulating STAT3 and cyclin-D1 expressions. Cancer Biol Ther. (2010) 10:386–90. 10.4161/cbt.10.4.12448 20562529

[24] Nishida N Arizumi T Takita M Kitai S Yada N Hagiwara S. Reactive oxygen species induce epigenetic instability through the formation of 8-hydroxydeoxyguanosine in human hepatocarcinogenesis. Dig Dis. (2013) 31:459–66. 10.1159/000355245 24281021

[25] Schneider M Weitz J. Acute oxygen deprivation and hypoxia tolerance. Dtsch Med Wochenschr. (2008) 133:2168–72. 10.1055/s-0028-1091258 18841523

[26] Mabjeesh NJ Amir S. Hypoxia-inducible factor (HIF) in human tumorigenesis. Histol Histopathol. (2007) 22:559–72. 17330811 10.14670/HH-22.559

[27] Ni J Wang X Stojanovic A Zhang Q Wincher M Bühler L. Single-Cell RNA sequencing of tumor-infiltrating NK cells reveals that inhibition of transcription factor HIF-1α unleashes NK cell activity. Immunity. (2020) 52:1075–87.e8. 10.1016/j.immuni.2020.05.001 32445619

[28] Hirota K Semenza GL. Regulation of angiogenesis by hypoxia-inducible factor 1. Crit Rev Oncol Hematol. (2006) 59:15–26. 10.1016/j.critrevonc.2005.12.003 16716598

[29] Semenza GL. Targeting HIF-1 for cancer therapy. Nat Rev Cancer. (2003) 3:721–32. 10.1038/nrc1187 13130303

[30] Ruan K Song G Ouyang G. Role of hypoxia in the hallmarks of human cancer. J Cell Biochem. (2009) 107:1053–62. 10.1002/jcb.22214 19479945

[31] Milano M Schneider M. EPO in cancer anemia: benefits and potential risks. Crit Rev Oncol Hematol. (2007) 62:119–25. 10.1016/j.critrevonc.2006.11.011 17197190

[32] Podolyak A Sessler DI Reiterer C Fleischmann E Akça O Mascha EJ. Perioperative supplemental oxygen does not worsen long-term mortality of colorectal surgery patients. Anesth Analg. (2016) 122:1907–11. 10.1213/ANE.0000000000001316 27195634

[33] Jiang Q Kurz A Zhang X Liu L Yang D Sessler DI. Supplemental intraoperative oxygen and long-term mortality: subanalysis of a multiple crossover cluster trial. Anesthesiology. (2021) 134:709–21. 10.1097/ALN.0000000000003694 33667304

[34] Kurz A Fleischmann E Sessler DI Buggy DJ Apfel C Akça O. Effects of supplemental oxygen and dexamethasone on surgical site infection: a factorial randomized trial. Br J Anaesth. (2015) 115:434–43. 10.1093/bja/aev062 25900659

[35] Wijesinghe M Perrin K Ranchord A Simmonds M Weatherall M Beasley R. Routine use of oxygen in the treatment of myocardial infarction: systematic review. Heart. (2009) 95:198–202. 10.1136/hrt.2008.148742 18708420

[36] Fonnes S Gögenur I Søndergaard ES Siersma VD Jorgensen LN Wetterslev J. Perioperative hyperoxia - Long-term impact on cardiovascular complications after abdominal surgery, a post hoc analysis of the PROXI trial. Int J Cardiol. (2016) 215:238–43. 10.1016/j.ijcard.2016.04.104 27128538

