# Supplementary material for: Intraoperative Fractions of Inspiratory Oxygen Are Associated With Recurrence-Free Survival After Elective Cancer Surgery
Source: Front Med (Lausanne). 2021 Nov 26;8:761786. doi: 10.3389/fmed.2021.761786 (PMC8661123; doi:10.3389/fmed.2021.761786)
Supplement: Supplementary file 1 [file Table_1.docx]

***Supplementary Material***

**Supplementary Table: Anesthesiologic and tumor-specific characteristics of the study cohort**

| **Variable** | **Analysis set (n=1084)** | **entity-controlled low-FiO_2_ (n=543)** | **entity-controlled high-FiO_2_ (n=541)** | ***p* value** |
| --- | --- | --- | --- | --- |

| Epidural anesthesia  CRC    PC    HC | n=405 60 (15) n=652 488 (75) n=27 20 (74) | n=203 21 (10) n=326 230 (71) n=14 10 (71) | n=202 39 (19) n=326 258 (79) n=13 10 (77) | **0.011  0.011** 0.745 |
| --- | --- | --- | --- | --- |
| Intraoperative dose of sufentanil (µg)  CRC  PC  HCC | n=405 84.30 ± 39.41 n=652 76.54 ± 47.35 n=27 70.19 ± 35.48 | n=203 88.45 ± 45.04 n=326 80.86 ± 51.16 n=14 73.21 ± 42.23 | n=202 80.12 ± 32.37 n=326 72.22 ± 42.85 n=13 66.92 ± 27.80 | **0.012  0.002** 1.000 |
| Intraoperative transfusion of RBC (TU)  CRC  PC  HCC | n=405 0.14 ± 0.62 n=652 0.29 ± 1.15 n=27 0.41 ± 1.37 | n=203 0.16 ± 0.67 n=326 0.21 ± 0.83 n=14 0.00 ± 0.00 | n=202 0.13 ± 0.56 n=326 0.38 ± 1.39 n=13 0.85 ± 1.91 | 0.839  0.427  0.325 |
| Intraoperative transfusion of FFP (TU)  CRC  PC  HCC | n=405 0.06 ± 0.47 n=652 0.27 ± 1.09 n=27 0.26 ± 0.94 | n=203 0.10 ± 0.60 n=326 0.27 ± 1.12 n=14 0.00 ± 0.00 | n=202 0.02 ± 0.28 n=326 0.26 ± 1.06 n=13 0.54 ± 1.33 | 0.058  0.773  0.519 |
| Intraoperative transfusion of PLT (TU)  CRC  PC  HCC | n=405 0.00 ± 0.00 n=652 0.02 ± 0.17 n=27 0.00 ± 0.00 | n=203 0.00 ± 0.00 n=326 0.02 ± 0.17 n=14 0.00 ± 0.00 | n=202 0.00 ± 0.00 n=326 0.02 ± 0.17 n=13 0.00 ± 0.00 | 1.000  1.000  1.000 |

| Total transfusion of RBC during entire hospital stay (TU)  CRC  0   1-5  6-10  11-15  > 15  PC  0  1-5  6-10  11-15  > 15  HC  0  1-5  11-15  > 15 | n=405 334 (83) 56 (14) 6 (2)  6 (2) 3 (1)  n=652 463 (71) 148 (23) 26 (4) 7 (1) 8 (1)  n=27 16 (60) 9 (33) 1 (4) 1 (4) | n=203 164 (81) 32 (16) 5 (3)  2 (1) 0 (0)  n=326 238 (73) 71 (22) 10 (3) 3 (1) 4 (19  n=14 10 (72) 4 (29) 0 (0) 0 (0) | n=202 170 (84) 24 (12) 1 (1)  4 (2) 3 (2)  n=326 225 (69) 77 (24) 16 (5) 4 (1) 4 (1)  n=13 6 (46) 5 (39) 1 (8) 1 (8) | 0.108  0.711  0.380 |
| --- | --- | --- | --- | --- |
| Total transfusion of PLT during entire hospital stay (TU)  CRC    PC    HC | n=405 0.03 ± 0.43 n=652 0.04 ± 0.35 n=27 0.04 ± 0.19 | n=203  0.00 ± 0.07 n=326  0.04 ± 0.38 n=14 0.00 ± 0.00 | n=202  0.06 ± 0.61 n=326  0.05 ± 0.31 n=13 0.08 ± 0.28 | 0.174  0.785  0.756 |
| Total transfusion of FFP during entire hospital stay (TU)  CRC  0  1-5  >5  PC  0  1-5  >5  HC  0  1-5  >5 | n=405 394 (97) 9 (2) 2 (1)  n=652 586 (90) 48 (7) 18 (3)  n=27 20 (74) 5 (19) 2 (7) | n=203 195 (96) 7 (3) 1 (1)  n=326 294 (90) 25 (8) 7 (2)  n=14 11 (79) 3 (21) 0 (0) | n=202 199 (99) 2 (1) 1 (1)  n=326 292 (90) 23 (7) 11 (3)  n=13 9 (69) 2 (15) 2 (15) | 0.245  0.613  0.306 |
| CRC  Open surgery, n (%) | n=405^a^ 362 (89) | n=145 123 (85) | n=260 239 (92) | **0.026** |
| UICC stage, n (%)  CRC  UICC 0-2  UICC 3-4  PC  UICC 0-2  UICC 3-4  HC  UICC 0-2  UICC 3-4 | n=404^b^ 236 (58) 168 (42)  n=652 650 (99.7) 2 (0.3)  n=6^b^ 3 (50) 3 (50) | n=145 84 (58) 61 (42)  n=391 390 (99.7) 1 (0.3) | n=259^b^ 152 (59) 107 (41)  n=261 260 (99.6) 1 (0.4)  n=6 3 (50) 3 (50) | 0.882  1.000 |
| Grading, n (%)  CRC  G1-2  G3-4  No grading possible after   neoadjuvant therapy  PC  G1-2  G3-4  No grading possible after   neoadjuvant therapy  HC  G1-2  G3-4  No grading possible after   neoadjuvant therapy | n=405 267 (66) 45 (11) 93 (23)  n=650 389 (60) 202 (31) 59 (9)  n=26 19 (73) 7 (27) 0 (0) | n=145 92 (63) 16 (11) 37 (26)  n=389 225 (58) 127 (33) 37 (10)  n=5 5 (100) 0 (0) 0 (0) | n=260 17 (67) 29 (11) 56 (22)  n=261 164 (63) 75 (29) 22 (8)  n=21 14 (67) 7 (33) 0 (0) | 0.653  0.444  0.278 |
| Resection margin status R1, n (%)  CRC  PC  HC | n=405 6 (2)  n=652 477 (73)  n=27 0 (0) | n=145 2 (1)  n=391 294 (75)  n=6 0 (0) | n=260 4 (2)  n=261 183 (70)  n=21 0 (0) | 0.899  0.152 |
| Tumor localization, n (%)^c^  CRC: Rectum  PC: pancreatic head | n=405 264 (65)  n=652 504 (77) | n=145 97 (67)  n=391 316 (81) | n=260 167 (64)  n=261 188 (72) | 0.589  **0.009** |
| Neoadjuvant chemotherapy, n (%)  CRC  PC  HC | n=405 82 (20)  n=652 79 (12)  n=27 1 (4) | n=145 34 (23)  n=391 52 (13)  n=6 0 (0) | n=260 48 (19)  n=261 27 (10)  n=21 1 (5) | 0.231  0.257  0.586 |
| Neoadjuvant radiotherapy, n (%)  CRC  PC  HC | n=405 111 (27)  n=652 45 (7)  n=27 0 (0) | n=145 41 (28)  n=391 24 (6)  n=6 0 (0) | n=260 70 (27)  n=261 21 (8)  n=21 0 (0) | 0.770  0.346 |
| IORT, n (%)  CRC  PC  HC | n=405 3 (1)  n=651 29 (4)  n=27 0 (0) | n=145 1 (1)  n=391 13 (3)  n=6 0 (0) | n=260 2 (1)  n=261 16 (6)  n=27 0 (0) | 1.000  0.089 |
| Adjuvant therapy, n (%)  CRC  PC  HC | n=328 145 (44)  n=553 488 (88)  n=27 1 (4) | n=119 59 (50)  n=335 302 (90)  n=6 0 (0) | n=209 86 (41)  n=218 186 (85)  n=21 1 (5) | 0.139  0.085  1.000 |

Data are presented as mean ± SD, or as absolute number (percentage). P-values refer to comparison between low-FiO_2_ vs. high-FiO_2_ patients. Continuous data were compared using Mann-Whitney U test. Categorial variables were compared using chi-square test. Boldface indicates p-values<0.05. ^a^ Only for colorectal cancer patients open versus laparoscopic surgery was differentiated; ^b^ UICC staging only possible for patients with known N status of the TNM classification; ^c^ Only for colorectal cancer patients (rectum versus colon) and pancreatic cancer patients (head versus other), tumor localization was determined.

FiO_2_: Fraction of inspired oxygen; SD: Standard deviation; UICC: Tumor classification according to the Union for International Cancer Control; CRC: Colorectal cancer; PC: Pancreatic cancer; HC: Hepatic cancer; IORT: Intraoperative radiation therapy; RBC: Red blood cells, FFP: Fresh frozen plasma, PLT: Platelet concentrates, TU: Transfusion units
